# Guanine content in the CTCF motif encodes a vulnerability to persistent, alcohol-induced redistribution

**DOI:** 10.64898/2026.10.14.759128

**Published:** 2026-10-16

**Authors:** Ben Nolan, Joynob A. Puspo, Iesha M. Querry-Peterson, Stephanie Maurina, Rebecca A. Wright, Allyson Schmitz, Lauren E. Jacobsen, Coralmarie Ortiz, Timothy E. Reznicek, Alexander Ethell, Hannah L. Harris, Christopher T. Cummings, M. Jordan Rowley

## Abstract

Alcohol consumption is a leading contributor to the global disease burden, yet its molecular consequences remain incompletely understood. Ethanol’s first metabolite, acetaldehyde, modifies guanine to form DNA adducts and DNA-protein crosslinks that block elongating RNA polymerase II. Because acetaldehyde acts specifically on guanine, we hypothesized that chromatin features would be disrupted in a sequence-dependent rather than stochastic manner. Here we confirm that alcohol exposure displaces elongating RNA polymerase II (RNAPIIs2p) from gene bodies, relocating to promoters. In addition to this confirmation, we found that RNAPIIs2p also relocates to discrete guanine-rich intergenic CTCF sites. Loci with high guanine content at the CTCF motif’s zinc finger 5 (ZF5) binding position showed the largest increase in both CTCF and cohesin (RAD21), with CTCF occupancy changes persisting even six days after alcohol withdrawal. Surprisingly, using Hi-C, we found that these changes to CTCF and cohesin do not impact the pronounced structural CTCF loops, but are instead primarily associated with altered guanine-rich enhancer-promoter contacts that connect to lasting changes to gene expression. Beyond our cell-line model, the same sequence rule accounts for altered CTCF occupancy in a mouse starvation (metabolic stress) model and in the placentae after paternal alcohol exposure. Together these findings link alcohol exposure to sequence-directed changes to chromatin whose rules are conserved in alcohol-associated disease.

**GRAPHICAL ABSTRACT:**

## Full Text Availability

The license terms selected by the author(s) for this preprint version do not permit archiving in PMC. The full text is available from the preprint server.

